# Preimplantation Genetic Diagnosis and Natural Conception: A Comparison of Live Birth Rates in Patients with Recurrent Pregnancy Loss Associated with Translocation

**DOI:** 10.1371/journal.pone.0129958

**Published:** 2015-06-17

**Authors:** Shinichiro Ikuma, Takeshi Sato, Mayumi Sugiura-Ogasawara, Motoi Nagayoshi, Atsushi Tanaka, Satoru Takeda

**Affiliations:** 1 Saint Mother Obstetrics and Gynecology Hospital, Fukuoka, Japan; 2 Department of Obstetrics and Gynecology, Juntendo University Graduate School of Medicine, Tokyo, Japan; 3 Department of Obstetrics and Gynecology, Nagoya City University, Graduate School of Medical Sciences, Nagoya, Japan; University of Florida, UNITED STATES

## Abstract

**Background:**

Established causes of recurrent pregnancy loss (RPL) include antiphospholipid syndrome, uterine anomalies, parental chromosomal abnormalities, particularly translocations, and abnormal embryonic karyotypes. The number of centers performing preimplantation genetic diagnosis (PGD) for patients with translocations has steadily increased worldwide. The live birth rate with PGD was reported to be 27-54%. The live birth rate with natural conception was reported to be 37-63% on the first trial and 65-83% cumulatively. To date, however, there has been no cohort study comparing age and the number of previous miscarriages in matched patients undergoing or not undergoing PGD. Thus, we compared the live birth rate of patients with RPL associated with a translocation undergoing PGD with that of patients who chose natural conception.

**Methods and Findings:**

After genetic counseling, 52 patients who desired natural conception and 37 patients who chose PGD were matched for age and number of previous miscarriages and these comprised the subjects of our study. PGD was performed by means of fluorescence in situ hybridization analysis. The live birth rates on the first PGD trial and the first natural pregnancy after ascertainment of the carrier status were 37.8% and 53.8%, respectively (odds ratio 0.52, 95% confidence interval 0.22-1.23). Cumulative live birth rates were 67.6% and 65.4%, respectively, in the groups undergoing and not undergoing PGD. The time required to become pregnancy was similar in both groups. PGD was found to reduce the miscarriage rate significantly. The prevalence of twin pregnancies was significantly higher in the PGD group. The cost of PGD was $7,956 U.S. per patient.

**Conclusions:**

While PGD significantly prevented further miscarriages, there was no difference in the live birth rate. Couples should be fully informed of the similarity in the live birth rate, the similarity in time to become pregnancy, the advantages of PGD, such as the reduction in the miscarriage rate, as well as its disadvantages, such as the higher cost, and the advantages of a natural pregnancy, such as the avoidance of IVF failure. The findings presented here should be incorporated into the genetic counseling of patients with RPL and carrying a translocation.

## Introduction

Established causes of recurrent pregnancy loss (RPL) include antiphospholipid syndrome (APS), uterine anomalies, parental chromosomal abnormalities, particularly translocations, and abnormal embryonic karyotypes [1–5]. In a meta-analysis covering 22,199 couples, De Braekeleer et al. found that the rate of chromosomal structural rearrangements in couples suffering from RPL was 4.7% [6].

The number of centers performing preimplantation genetic diagnosis (PGD) has steadily increased worldwide since the procedure was first introduced over twenty years ago [7]. PGD by fluorescence in situ hybridization (FISH) analysis was initiated in 1998 as a means of preventing miscarriages in patients with RPL associated with a translocation [8]. The live birth rate in patients undergoing PGD was reported to be in the range of 27–54%, while that on the first pregnancy after ascertaining the carrier status ranged from 37–63% in patients who chose to conceive naturally [9–12]. The cumulative live birth rate for patients in the later group was 65–83% [2, 13–16].

Fischer et al. concluded that PGD could significantly lower the miscarriage rate from 88.5% to 13% in translocation carriers [12]. However, as most RPL patients seek medical advice only after they experience difficulty in having children, it would be inappropriate to simply compare miscarriage rates before and after a diagnosis of RPL. With respect to this, conclusions of an article by Fischer et al. were refuted by Stephenson and Goddijn [17].

No laws have been passed on the use of PGD in Japan. However, for ethical reasons, the Japan Society of Obstetrics and Gynecology (JSOG) initially established a policy limiting PGD to patients suffering from extremely severe genetic diseases. PGD for patients with RPL associated with a translocation has been permitted from December 2006.

The present study was therefore conducted to compare the live birth rates of patients with RPL associated with a translocation who underwent PGD and those of non-PGD patients who chose natural conception instead. To the best of our knowledge, this is the first comparison of the live birth rates of patients in which both groups have been matched for age and the number of previous miscarriages.

## Materials and Methods

### Patients

A total of 126 patients with a history of RPL (two or more consecutive clinical miscarriages) associated with a translocation were enrolled. All patients were seen at Saint Mother Obstetrics and Gynecology Hospital or Nagoya City University Hospital between August 2003 and November 2013, for investigation of the cause of the RPL or genetic counseling. All patients underwent a systematic examination, including hysterosalpingography, chromosome analysis of both partners, diagnostic tests for APS, including screening for lupus anticoagulant by aPTT and dilute Russell’s viper venom time and (βeta2 glycoprotein I-dependent) anticardiolipin antibody, and blood tests for hypothyroidism and diabetes mellitus, before a subsequent pregnancy.

**Fig 1:**
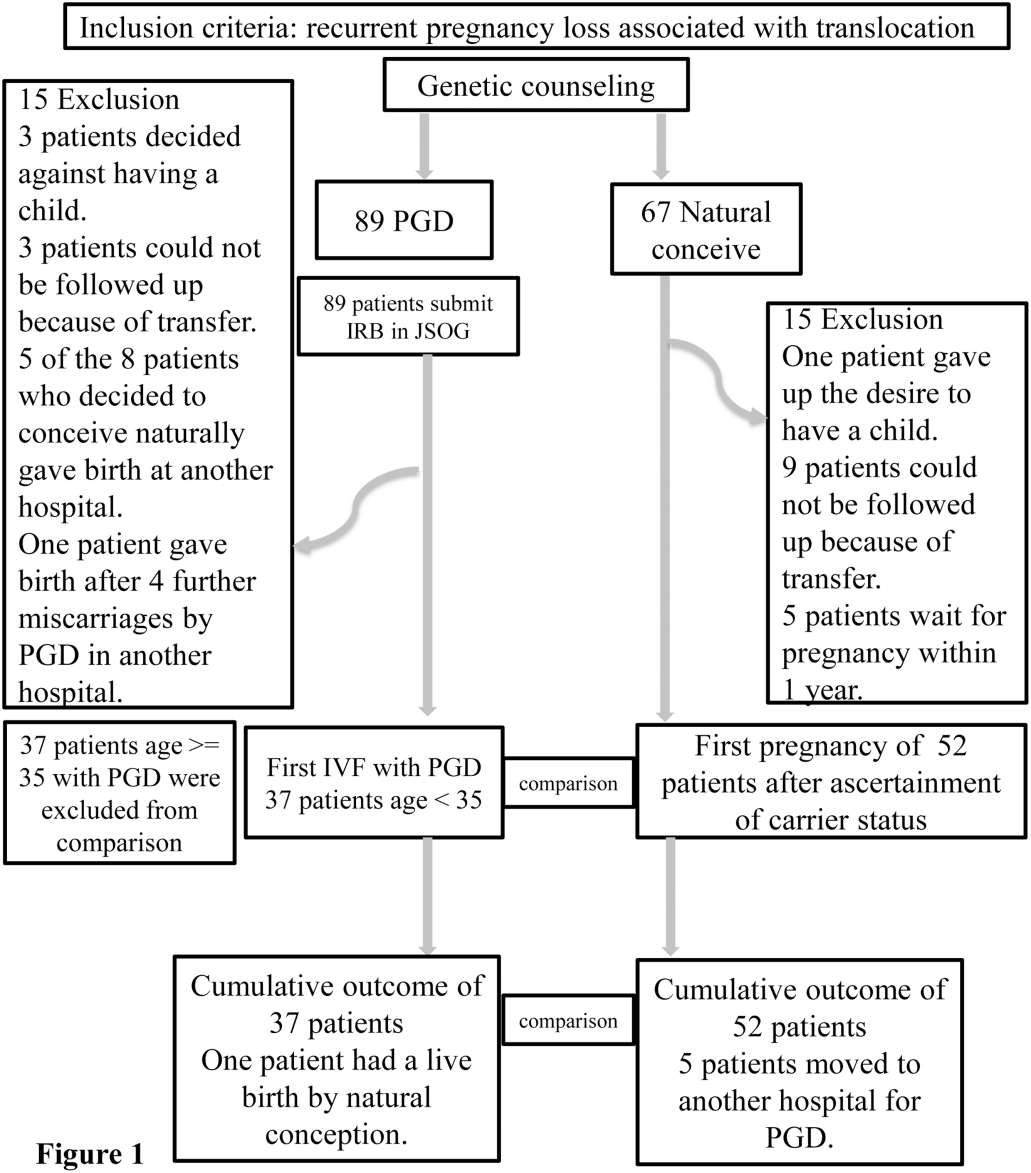
Of 156 patients with reciprocal or Robertsonian translocations who received genetic counseling, 67 chose natural conception and 15 were excluded from the study. The remaining 89 chose PGD and 15 were excluded from the study. All comparisons were performed between the 37 patients of the PGD group who were ≤34 years old and the 52 patients who conceived naturally so that the patients who underwent PGD could be matched for age. The subsequent outcomes of the 126 patients were ascertained from the medical records and by telephone until July 2014.

Of 156 patients with reciprocal or Robertsonian translocations who received genetic counseling, 30 were excluded from the study (Fig 1).

Among the 126 patients enrolled, 52 chose natural conception and were followed up. These 52 patients were seen at Nagoya City University Hospital. One patient decided against becoming pregnant after receiving genetic counseling. Nine patients relocated and could not be followed up by telephone or mail. Five patients were still trying to become pregnant within 1 year of enrollment in the study.

The remaining 74 of the 126 patients underwent PGD. All patients were seen at Saint Mother Obstetrics and Gynecology Hospital or Nagoya City University Hospital. Three patients decided against bearing a child. Three patients relocated and could not be followed up by telephone or mail. Five of 8 patients who changed their mind and decided to conceive naturally gave birth at another hospital. One patient underwent PGD at another hospital.

The subsequent outcomes of the 126 patients were ascertained from the medical records and by telephone until July 2014.

### Ethics statement

This study was conducted with the approval of the Research Ethics Committee of Nagoya City University Graduate School of Medical Sciences. Each patient wishing to undergo PGD had their case reviewed by the Research Ethics Committee of Saint Mother Obstetrics and Gynecology Hospital and Nagoya City University Graduate School of Medical Sciences, and each case was then reviewed the Ethics Committee of the JSOG and permission granted. Patients provided their written informed consent to participate in this study. However, the karyotype of 7 patients whose informed consent to publication of their medical data could not be obtained was not described in Table 1.

**Table 1:** Carrier status of the 52 patients who conceived naturally.

Carrier status	M	S	L	age	Subsequent pregnancies
45,XX,der(13;14)(q10;q10)	4	0	1	37	SA(46,XX,der(13;14)(q10;q10),+16), T
45,XX,der(13;14)(q10;q10)	4	0	0	33	T
45,XX,der(13;14)(q10;q10)	3	0	0	33	T
45,XX,der(13;14)(q10;q10)	2	0	0	28	T, preterm(36w)
45,XX,*	2	0	0	31	EP
45,XX,der(13;21)(q10;q10)	5	0	1	30	SA
45,XX,der(14;15)(q10;q10)	2	0	0	29	T(46,XX)
45,XX,der(15;22)(q10;q10)	3	0	0	33	T
45,XX,*	2	0	0	35	SA(Tetraploidy)
45,XY,der(13;14)(q10;q10)	3	0	0	31	T
45,XY,der(14;15)(q10;q10)	2	0	0	37	T(47,XY,+21), T
45,XY,der(21;22)(q10;q10)	7	0	0	32	SA
45,X?,t(13;14)	3	0	1	31	SA(68,XXY,der(13;14)(q10;q10)), T
46,XX,t(1;13)(q41;q22)	4	0	0	23	SA(46,XY,der(13)t(1;13)(q41;q22)mat)
46,XX,t(1;21)(p34.1;q22.1)	3	0	0	30	T
46,XX,t(1;3)(p36.1;p21.3)	3	0	0	30	T(46,XX)
46,XX,t(10;19)(q24.3;q13.4)	2	0	0	29	T
46,XX,t(11;13)(q22.2;q14.1)	3	0	0	34	T
46,XX,t(13;18)(q14.3;q21.3)	3	1	0	31	preterm(36w,46,XX),SA(46,XX,der(18)t(13;18)(q14.3;q21.3)mat), T(46,XX)
46,XX,t(13;22)(q32;q11.2)	4	0	1	33	T
46,XX,t(15;17)(q11.2;q21.1)	2	0	0	28	No conception
46,XX,t(2;22)(p22;q13.2)	4	0	0	32	SA, SA
46,XX,t*	4	0	0	31	IUFD(14w,46,XX,conjoined twin), SA, T
46,XX,t*	2	0	0	26	IUFD(14w,unbalanced)
46,XX,t(6;7)(q15;q22)	2	0	0	35	T(46,XX,t(6;7)(q15;q22))
46,XX,t(6;7)(q23.3;q34)	1	1	0	35	SA(46,XY,der(6)t(6;7)(q23.3;q34)), SA, SA
46,XX,t(7;13)(q33;q21.3)	4	0	1	27	SA(47,XY,+der(13)t(7;13)(q33;q21.3)mat[28]/ 48,XXY,+der(13)t(7;13)(q33;q21.3)mat[2]), SA, T
46,XX,t*	3	0	0	28	SA, SA
46,XY,t(1;10)(p34;p13)	3	0	0	37	SA(46,XX)
46,XY,t(10;17)(q11.2;p11.2)	3	0	1	33	T
46,XY,t(12;14)(q24.1;q32)	4	0	0	27	preterm(36w,twin)
46,XY,t(12;15)(q24.31;q11.2)	3	0	1	34	T
46,XY,t(13;14)(q14.3;q32.1)	3	0	0	24	SA(46,XY,der(14)t(13;14)(q14.3;q32.1)pat
46,XY,t(13;15)(q14.1;q26.1)	3	0	0	31	SA(46,XX,der(13)(13;15)(q14.1;q26.1))
46,XY,t(2;16)(q37.1;q24)	2	0	0	27	T, T, SA
46,XY,t(2;7)(q31;q34)	3	0	0	26	T, T, SA
46,XY,t(4;15)(p15.3;q25)	3	0	0	35	SA(46,XY,der(4)t(4;15)(p15.3;q25)pat)
46,XY,t(4;19)(q22;p13.3)	2	0	0	34	SA, T
46,XY,t(5;13)(q21;q12.1)	2	0	0	29	No conception
46,XY,t(5;8)(q23.1;p23.2)	4	1	0	32	T
46,XY,t(6;15)(q23;q21.1)	3	0	0	28	SA(46,XX,add(6)(p23)), BP, T
46,XY,t(6;8)(p11.2;p21)	3	0	1	34	T
46,XY,t*	3	0	0	27	T
46,XY,t*	2	0	0	27	T
46,XY,t(7;8)(q31.2;q24.22)	3	0	0	32	SA(46,XX)
46,XY,t(10;14)(q25.2;q22)	3	0	0	29	SA, SA
46,X?,t(1;4)(q23.1;p15.3)	1	1	0	34	T
46,XX,t(4;12)(q35;q13.3), 46,XY,?t(Y;6)(q12;q27)	3	0	0	32	T
46,XX,der(3)(3pter-3q13.3::16q22-16qter),der(16)(16pter-q22::3q24-3q13.3::3q24-3qter)	2	0	0	32	T
46,XX,t(1;17;13;8)(q32.1;q21.1;q22;p22)	4	0	0	31	SA(47,XX,der(17)(17pter→17q21.1::1q32.1→1qter)mat,+der(13)(13pter→13q22.1::17q21.1→17qter)mat.ish der(17)(17pter→17q21.1::1q32.1→1qter)(GS-160H23+,GS-50C4-),+der(13)(13pter→13q22.1::17q21.1→17qter)(GS-163C9-,GS-50C4+,wcp13+)
46,XY,der(4pter-4q22::17q23-17qter)der(17)(17q23::?::4q25-4qter)	3	0	0	40	T(46,XX,t(4;17)(q21;q23))
46,XY,t(1;4;15)(q44;p15.2;q26.1)	5	1	0	21	T, T

*Patients whose informed consent could not be obtained,

M: prior miscarriage, S: prior stillbirth, L: prior live birth, SA: spontaneous abortion, T: term delivery, IUFD: intrauteriine fetal death, EP: ectopic pregnancy, BP: biochemical pregnancy.

### Comparison and Statistical Analysis

The mean (SD) age at the ascertainment of the translocation and the numbers of previous pregnancy losses, stillbirth rates and live birth rates were compared by Student’s t-test between patients who did/did not undergo PGD. Male: female and reciprocal: Robertsonian ratios of the two groups were compared by the chi-square test. Patients who were not able to conceive for more than 1 year were determined to have an infertility problem.

The mean age of the patients who underwent PGD was significantly higher than that of the patients who conceived naturally. Thus, the comparison of the two groups was carried out using patients aged < = 34 years.

The live birth rates on the first PGD trial and the first natural pregnancy after ascertaining the carrier status were compared by the chi-square test. Cumulative live birth rates were also compared. Comparisons were also made of the mean number of further miscarriages, the mean number of months (duration) from the day of establishing the carrier status to the day of ascertainment of a live birth pregnancy, the frequency of babies with congenital anomalies and the number of twin pregnancies.

PGD was initiated in December 2006 because the JSOG did not permit PGD prior to that year, and this represented the first use of PGD in Japan. Thus, the duration for patients who desired PGD before December 2006 was calculated from the day permission was granted by the JSOG until a successful pregnancy.

The mean number of oocyte retrievals and embryo transfers and the total cost of PGD were calculated for the patients who underwent PGD.

All analyses were carried out using the statistical software SPSS, Version 21. P < 0.05 was considered to denote statistical significance.

### Methods for preimplantation genetic diagnosis

PGD by means of FISH analysis was performed after IVF on blastomeres obtained from day-3 embryos at about the 8-cell stage. The embryos were cultured as described by Tanaka et al [18]. When the single interphase nucleus of a blastomere was observed, the blastomeres were biopsied according to a manipulation technique described by Takeuchi et al [19].

The biopsied blastomeres were washed with phosphate-buffered saline (PBS) and exposed to hypotonic solution (0.075 mol/l KCl), and were then fixed and attached on glass slides. The fixed blastomeres were subjected to FISH analysis. The strategy consisted of using two subtelomeric probes for both translocation-related chromosomes and one centromeric probe for either translocation-related chromosome. The probes were obtained from Vysis (USA), Cytocell (UK) and GSP laboratory (Japan), and labeled in different colors. The nuclei and probe mixture were combined on the glass slides and denatured by heating to 72°C. The slides were then incubated in a chamber overnight at 37°C to allow for hybridization. The slides were analyzed using a fluorescence microscope (IX71, Olympus, Japan). Blastomeres showing two signals for each probe were classified as normal or balanced, while any other combination was classified as unbalanced.

Up to two embryos with normal or balanced FISH signals were selected and transferred into the uterine cavity on day 5 after the IVF or vitrified and transferred in the thaw cycle depending on the condition of the embryo and the patient.

## Results

All comparisons were performed between the 37 patients of the PGD group who were ≤34 years old and the 52 patients who conceived naturally so that the patients who underwent PGD could be matched for age (Tables 1 and 2). A total of 89 couples had a translocation in one partner, 36 in men and 49 in women. In one couple, a reciprocal translocation was found in both partners. Four couples had the complex reciprocal type of translocation. Of all the translocations, 71 were reciprocal, and 18 were Robertsonian. Patient characteristics are shown in Table 3.

**Table 2:** Carrier status of the 37 patients who underwent PGD.

Carrier status	M	S	L	age	OR	ET	Subsequent pregnancies
45,XX,der(13;14)(q10;q10)	4	0	0	32	2	2	T(twin)
45,XX,der(15;21)(q10;q10)	5	0	0	27	3	3	EP
45,XY,der(13;14)(q10;q10)	2	1	0	31	1	1	T
45,X?,der(14;21)(q10;q10)	3	0	0	27	1	1	T, T
46,XX,t(1;13)(q41;q22)	5	0	0	23	1	1	T, T
46,XX,t(10;16)(p11.2;q13)	3	0	0	34	3	2	T
46,XX,t(10;21)(q26;q21)	5	0	0	32	2	2	EP
46,XX,t(11;22)(q23.3;q11.2)	2	0	0	34	6	6	No conception
46,XX,t(19;20)(q13.4;p11.2)	3	0	0	32	1	1	T
46,XX,t(2;13)(p25.3;q14.1)	3	1	0	33	2	1	No conception
46,XX,t(2;21)(q32.1;q11.2)	2	0	0	34	10	6	SA(47,XX,+22), T
46,XX,t(2;5)(p25.1;q35.1)	5	0	0	30	9	8	termination (47,X?,+21)
46,XX,t(2;8)(q11.2;p23)	3	0	0	31	2	3	T(twin)
46,XX,t(3;21)(q13.2;q22.3)	2	1	0	30	1	1	T
46,XX,t(3;8)(q13;q12)	4	0	1	33	1	1	No conception
46,XX,t(4;8)(p14;q22.3)	2	0	0	34	1	2	BP, T
46,XX,t(6;10)(q23.3;q25.2)	3	0	1	32	4	4	SA
46,XX,t(6;7)(q21;p22)	3	0	0	34	1	1	T, T(twin)
46,XX,t(7;18)(p11.2;p11.2)	2	0	1	31	3	3	preterm(36w,twin)
46,XX,t(7;8)(q11.2;q13)	4	0	0	31	1	1	preterm(36w,twin)
46,XX,t(7;8)(q21.1;p23.1)	4	0	0	31	2	2	BP
46,XX,t(7;8)(q31.3;q13)	3	0	0	30	1	1	T(46,XX,t(7;8)(q31.3;q13)mat)
46,XX,t(8;12)(q24.1;p13.1)	5	0	0	34	1	1	No conception
46,XX,t(8;13)(q24.1;q14.1)	2	0	0	27	3	1	T
46,XX,t(8;22)(q24.1;q11.2)	3	0	0	28	2	2	BP, preterm(36w,twin)
46,XY,t(1;5)(q36.1;q31)	2	0	0	29	1	1	T, T
46,XY,t(12;17)(q24.3;q21.3)	3	0	1	33	1	1	No conception
46,XY,t(13;14)(q22;q11.2)	2	0	0	32	1	1	T
46,XY,t(2;18)(p24;q11.2)	2	0	0	26	6	2	No conception
46,XY,t(2;22)(q11.1;p13)	2	0	0	26	1	1	T, T(twin)
46,XY,t(2;5)(q13.1;q35.1)	6	0	0	33	3	4	SA, T
46,XY,t(4;11)(q31.2;p15.3)	3	0	0	26	1	1	T, SA(47,XY,+2,t(4;11)(q31.2;p15.3)pat), preterm (25w,twin)
46,XY,t(6;18)(q15;q22)	3	0	1	32	1	2	T
46,XY,t(7;11)(q11.23;q12)	7	0	0	34	7	7	preterm(36w, twin)
46,XY,t(7;8)(q32.3;q32)	4	0	0	26	1	1	T
46,XY,t(9;13)(q12;q13)	4	0	0	28	3	1	T(natural)
46,X?,t(6;13)(q25.1;q31)	2	0	0	34	1	1	No conception

M: prior miscarriage, S: prior stillbirth, L: prior live birth, OR: the cycles of oocyte retrieval, ET: cycles of embryo transfer.

SA: spontaneous abortion, T: term delivery, IUFD: intrauterine fetal death, EP: ectopic pregnancy, BP: biochemical pregnancy.

**Table 3:** Characteristics of the 89 patients with a history of recurrent pregnancy loss who underwent PGD or conceived naturally.

	Patients with PGD	Patients who conceived naturally	P-value
No. of patients	37	52	
Mean age (SD)	30.6 ± 3.0	30.9 ± 3.8	NS
Male: female	12: 23	23: 26	NS
Reciprocal: Robertsonian	33: 4	38: 14	NS
Complex translocation	0	4	
Translocation in both partner	0	1	
Mean (SD) No. of previous pregnancy losses	3.37 ± 1.26	3.10 ± 1.07	NS
2	10	16	
3	13	22	
4	7	10	
5	5	2	
6	1	1	
7	1	1	
No. of previous still births	0.08 ± 0.28	0.10 ± 0.30	NS
0	34	47	
1	3	5	
2	0	0	
No. of previous live births	0.14 ± 0.35	0.15 ± 0.36	NS
Primary	32 (86.5%)	44 (84.6%)	
Secondary	5 (13.5%)	8 (15.4%)	
Presence of infertility with IVF	6 (16.2%)	6 (11.5%)	NS

The mean (SD) age and number of previous miscarriages in the PGD group and natural conception group were 30.6 (3.0) vs. 30.9 (3.8), and 3.37 (1.26) vs. 3.10 (1.07), respectively (not significantly different). There were also no significant differences between the two groups in the male/female or reciprocal/Robertsonian translocation ratio, the percentage of infertile patients, or the mean number of previous stillbirths and live births.

The live birth rates on the first PGD trial and the first pregnancy after ascertainment of the carrier status were 37.8% and 53.8%, respectively (Table 4). Thus, there was no difference in the live birth rate when PGD and natural conception were compared (odds ratio (OR) 0.52, 95% confidence interval (CI) 0.22–1.23, p = 0.101). The cumulative live birth rates in the two groups were 67.6% and 65.4% (OR 1.10, 95%CI 0.45–2.70).

**Table 4:** Subsequent live birth rate in patients who underwent PGD or conceived naturally.

	37 patients aged < = 34 years who underwent PGD	52 patients who conceived naturally	OR (95% CI)*	p-value
Live birth rate on the first trial	37.8% (14/37)	53.8% (28/52)	0.52 (0.22–1.23)	0.10
Cumulative live birth rate	67.6% (25/37)	65.4% (34/52)	1.10 (0.45–2.70)	0.83
Infertility	18.9% (7)	3.8% (2)	**1.19 (1.00–1.40)**	**0.03**
Total (range) and mean number of further miscarriages until a live birth	9 (0–1) and 0.24 ± 0.40	30 (0–3) and 0.58 ± 0.78	-	**0.02**
Biochemical pregnancy*	1	1		
Ectopic pregnancy*	2	1		
Mean number of oocyte retrievals	2.46 (2.30)	-		
Mean number of embryo transfers	2.16 (1.85)	-		
Mean (SD) months from genetic counseling until successful pregnancy	12.4 (13.95)	11.4 (10.9)	NS	
Congenital anomaly	1**	1		
Twin pregnancy/live birth	29.0% (9/31) at 25w (1), 36w (4), 37w (3), 38w (1)	5.1% (2/39) at 36w (2)	**7.57 (1.50–38.26)**	**0.009**
Cost/ patient	$7,956 U.S.***	-		

*Biochemical and ectopic pregnancies were included.

**A fetus with 21 trisomy was terminated at 18 weeks’ gestation.

***The cost is speculated to be lower. The cost ranged from $8,000–10,000 U.S. per trial in other hospitals in Japan. A technical charge was not included in the cost because this study was conducted for clinical research.

The mean number of further miscarriages untill a live birth took place in the PGD (0.22 ± 0.42) group was significantly lower than in the natural conception group (0.58 ± 0.78, p = 0.012). Thus, PGD significantly reduced the miscarriage rate.

There were no differences in the two groups in the mean interval of months (SD) from genetic counseling to ascertainment of a pregnancy ending in a live birth (12.4 vs. 11.4). In patients of the PGD group, the mean number of oocyte retrievals was 2.46 and the mean number of embryo transfers was 2.16.

A congenital anomaly was found in one case of the PGD group (pregnancy was terminated at 18 weeks’ gestation in a woman carrying a fetus with trisomy 21), and in one case of the natural conception group (a newborn baby with Down syndrome) (Tables 1 and 2). The abnormality was not derived from the parental Robertsonian translocation. The twin pregnancy/live birth ratio was significantly higher in the PGD group than in the natural conception group (OR 7.57, 95% CI 1.50–38.26). Cesarean section was performed in one patient at 25 weeks’ gestation, and the body weights of the twins were 744 g and 782 g.

The mean cost of PGD per patient was 961,667 yen ($7,956 U.S. at $ equal to 120.9 yen, January, 2015).

The live birth rate on the first trial (13.5%, 5/37) and the cumulative live birth rate (24.3%, 9/37) of the PGD group who were > = 35 years old were significantly lower than those of the PGD group who were ≤34 years old (OR 0.26, 95%CI 0.08–0.9–1 and OR 0.15, 95%CI 0.06–0.43).

## Discussion

Our present study proved that there was no difference in the live birth rates of the PGD and natural conception groups.

Since 1998, PGD has been though to improve the incidence of live births and 4253 cycles have been performed around the world [8, 10, 20]. However, no cohort study to evaluate the live birth rate has been conducted. The live birth rate in patients with PGD was reported to be in the range of 27–54% [9–12], while in the patients who conceived naturally, the range was 37–63% at the first pregnancy after ascertainment of the carrier status [2, 13–16]. A simple comparison would be difficult because the age and number of previous miscarriages differed among the studies. Meta-analyses have also concluded that there are insufficient data to show that PGD improves the live birth rate in couples with RM carrying a structural chromosome abnormality [21]. In the present study, the mean age of patients who desired PGD was significantly higher. Thus, our comparison was carried out after the patients aged > = 35 years were excluded from the PGD group. This may be one of the limitations of the present study.

However, PGD did reduce the miscarriage rate significantly. The reported miscarriage rates in patients with PGD range from 0–10.2% [9–12], while those in patients undergoing natural conception are in the range of 37–62% [2, 13–16]. Since we selected and transferred embryos with normal or balanced FISH signals, we assumed that miscarriages after PGD were not caused by translocation-related chromosomes, but rather by aneuploidy. Thus, there are several technical limitations to PGD using FISH analysis for detecting two translocation-related chromosomes. To overcome this, it would be necessary to perform screening of the blastocyst trophectoderm for all 24 types of chromosomes by mean of array comparative genomic hybridization (CGH). However, preimplantation genetic screening (PGS) is not permitted for ethical reasons in Japan.

The live birth rate and miscarriage rate in patients with RPL associated with a translocation were 50.0% and 0% in Fiorentino’s study in which CGH was used [22]. CGH might be superior to FISH in reducing the number of miscarriages, although a simple comparison might be difficult because of the differences in patient characteristics. CGH might have prevented the cases of fetal trisomy in the present study (Table 2), but it is unclear whether CGH would have improved the live birth rate.

Several randomized controlled trials (RCT) have demonstrated that PGS with comprehensive chromosome screening or FISH can increase the live birth rates in groups with a good prognosis [23–25]. The inclusion criteria in those studies consisted of younger women, lower serum FSH levels, a higher number of retrieved oocytes, no more than one failed IVF, and no more than one miscarriage. PGS might be a good method for selecting the best of many embryos in patients with a good prognosis and allow for delivery one or two months earlier than with natural conception. PGS might have no benefit in patients with a poor prognosis who cannot give birth by natural conception.

Scott et al. proved that the live birth rate with trophectoderm biopsy was superior to that with cleavage stage biopsy though their sample size was relatively small [26]. Use of a cleavage stage biopsy might be one of the reasons why the pregnancy rate decreased in an RCT comparing PGS+IVF and IVF alone [27]. On the other hand, the improvement in the live birth rate of patients with an advanced maternal age was shown by PGS with the use of both a day 3 biopsy and FISH [24].

Further RCTs incorporating new technologies should be implemented as soon as possible. For over 15 years, PGD/PGS using older technology has been performed worldwide without ever establishing that PGD improved the live birth rate. Several researchers including us speculated that PGD could not improve the live birth rate though it might initially reduce the miscarriage rate [28]. The present study is important because this is the first comparison of the live birth rate in patients with RPL associated with a translocation. It was worth nothing that none of the previous studies employed controls [9–12, 22].

Three reports have indicated that IVF-PGD causes multiple pregnancies. Lim et al. reported one case of a preterm twin delivery at 26 weeks of gestation because of an incompetent internal os as well as 2 cases of twin deliveries at term [9]. Feyereisen et al. reported 5 cases of twin pregnancies and one case of triplet pregnancies [11]. Fiorentino et al. reported 2 twin pregnancies that had completed at least 20 weeks of gestation [22]. Thus, because PGD is associated with a higher risk of multiple pregnancies, the risk of preterm delivery is also higher. Single embryo transfer should therefore be selected.

Indeed, an RCT is more appropriate for comparing the live birth rates. However, that would seem impossible in Japan, because each case must be submitted to the Ethics Committee of the JSOG. Another limitation of this study was that the prognosis in women aged > = 35 years remained unclear. It remains unestablished what chromosome numbers and translocation breakpoints might be risk factors. Thus, there might be bias between two groups. This was another limitation of the study.

In summary, couples should be fully informed of the similarities in the live birth rate and the time needed to become pregnant, the advantages of PGD such as reduction in the miscarriage rate, the disadvantages of PGD such as the higher cost, and the advantages of a natural pregnancy such as the avoidance of IVF failure. Amniocentesis may be considered for detecting any fetal abnormalities derived from translocations in patients choosing to conceive naturally, although in the present study, there was no case of any woman choosing to experience natural pregnancy who showed any abnormality of the fetus related to the translocation. These data should be incorporated into the genetic counseling of patients with RPL and a translocation.
